# Catalytic Ketonization over Oxide Catalysts (Part XIV): The Ketonization and Cross-Ketonization of Anhydrides, Substituted Acids and Esters †

**DOI:** 10.3390/molecules29030584

**Published:** 2024-01-24

**Authors:** Marek Gliński, Małgorzata Gidzińska, Łukasz Czerwiński, Kasper Drozdowski, Ewa M. Iwanek (nee Wilczkowska), Andrzej Ostrowski, Dariusz Łomot

**Affiliations:** 1 Faculty of Chemistry, Warsaw University of Technology, Noakowskiego 3, 00-662 Warsaw, Poland; lukasz.czerwinski@mixbox.pl (Ł.C.); kasper.drozdowski@gmail.com (K.D.); ewa.iwanek@pw.edu.pl (E.M.I.); andrzej.ostrowski@pw.edu.pl (A.O.); 2 Institute of Physical Chemistry, Polish Academy of Sciences, Kasprzaka 44/52, 01-224 Warsaw, Poland; dlomot@ichf.edu.pl

**Keywords:** ketonization, anhydrides, carboxylic acids, esters, ketones, metal oxide catalysts

## Abstract

A series of 20 wt.% MO_2_/S catalysts (where M = Ce, Mn or Zr and S = SiO_2_ or Al_2_O_3_) were prepared using various precursors of the active phases. The resulting catalysts were characterized using different methods (XRD, TPR and S_BET_). For the first time, anhydrides were used as potential starting materials for ketone synthesis. This novel reaction was performed on various aliphatic anhydrides in the presence of catalysts within a temperature range of 523–723 K. For all anhydrides, except for pivalic anhydride, the appropriate ketones were obtained with good or very good yields. The vapor-phase catalytic ketonization of esters of benzene-1,x-dicarboxylic acids (x = 2, 3 or 4) with acetic acid were studied in the range of 673–723 K in order to obtain 1,x-diacetylbenzenes. Their yields strongly increased with an increase in the x value (0, 8 and 43% for x = 2, 3 and 4, respectively). The presence of acetophenone as a side product was always noted. In the case of ω-phenylalkanoic acids, their vapor-phase ketonization with acetic acid led to the formation of appropriate ketones with 47–49% yields. Much lower yields of ketones (3–19%) were obtained for acids and ethyl esters containing heterocycle substituents (with O or S atoms) and/or vinyl groups. In the reaction between ethyl 4-nitrophenylacetate and acetic acid, only the products of ester decomposition (*p*-toluidine and *p*-nitrotoluene) were determined.

## 1. Introduction

Various carbonyl compounds are of importance as raw materials for organic synthesis, and many synthesis methods have been developed for their preparation. The oldest method of ketone synthesis is based on the pyrolysis of metal carboxylates. It has been known since 1852 [1], and it is carried out via Equation (1). Aldehydes can be prepared via the pyrolysis of a mixture of salts from formic and other carboxylic acids [2], as shown in Equation (2). The pyrolysis of metallic salts from dicarboxylic acids, as proposed by Ružicka et al. in the 1920s, has enabled the formation of cycloalkanones containing up to 32 carbon atoms in the ring [3], as shown in Equation (3). In 1895, a significant improvement in the synthesis of carbonyl compounds via a ketonization reaction was reported [4]. This development was the first continuous method of synthesis. The method shown in Equation (4) consists of passing the vapors of pure carboxylic acids through a catalyst bed at a temperature of around 723 K. When two different acids react, three ketones, two symmetric and one non-symmetric, are formed, as shown in Equation (5). The catalytic oxidation of alcohols [5,6,7,8] and other interesting ways of carrying out the synthesis of ketones with heterogeneous catalysts have also been studied [9,10,11,12].

(RCOO)_2_M → R_2_C=O + MCO_3_ (1)

(RCOO)_2_M + (HCOO)_2_M → 2RCH=O + 2MCO_3_ (2)

[(CH_2_)_n_(COO)_2_]_2_M → cyclo (CH_2_)_n_C=O + M(CO_3_)_2_ (3)

2RCOOH → R_2_C=O + CO_2_ + H_2_O (4)

RCOOH + R’COOH → ½(1 − x) R_2_C=O + x RR’C=O + ½(1 − x) R’_2_C=O + CO_2_ + H_2_O (5)

where x = <0, 1>.

When the use of a free acid is inconvenient or not feasible, aliphatic esters may be employed as precursors of these acids [13,14], as shown in Equation (6). This method has been applied for the synthesis of many types of ketones and aldehydes. A broad range of carboxylic acids, such as straight-chained, branched, cyclic or even unsaturated ones, have been transformed into ketones [15,16,17,18]. The oxides of manganese, cerium, zirconium and thorium deposited onto the surface of the supports (Al_2_O_3_, SiO_2_, TiO_2_, active carbon or pumice) are active catalysts [18]. Manganese and cerium oxides, due to the possibility of changing the oxidation states of manganese and cerium, have been commonly applied as catalysts [19,20,21,22] and catalyst supports [23,24], even in combination [25]. ZrO_2_ has also been applied in catalysis for decades [26,27].

2RCOOC_2_H_5_ → R_2_C=O + 2C_2_H_4_ + CO_2_ + H_2_O (6)

A few mechanisms for this reaction have been proposed in the literature. According to Bamberger’s mechanism, two molecules of an acid react with each other, leading to the formation of an anhydride, which decarboxylates into a ketone molecule in the next step [28]. In contrast, Neunhoffer and Paschke have postulated that the main step of the ketonization reaction begins with the formation of a β-keto acid intermediate that, due to its thermal instability, decomposes into ketone and carbon dioxide [29]. The mechanism proposed by Kwart and King explains the occurrence of the reaction with acids that lack an α-hydrogen atom, such as benzoic acid [30]. The authors assumed that in the crucial step, an alkyl or aryl anion is formed, whose presence is responsible for the subsequent steps to be carried out, leading to the formation of a ketone molecule. Recently, the ketonization reaction and its mechanism and scope have been reviewed [31].

Currently, high-molecular-weight ketones obtained during the ketonization reaction of carboxylic acids are applied as components in the production of diesel fuel. Moreover, in the Mix-Alco process, biomass and municipal and agricultural waste undergo biotransformation, leading to the formation of carboxylic acids, which are transformed into a mixture of heavy secondary alcohols known as co-solvents in the subsequent steps (ketonization and hydrogenation), increasing the solubility of ethanol in gasoline.

The novel method of synthesizing ketones from the anhydrides of carboxylic acids can be successfully implemented in the ketonization reaction. The scope of the application of this method has been determined. The idea stemmed from Bamberger’s mechanism concerning the formation of an anhydride molecule in the first step of the ketonization reaction of free carboxylic acids. This method is used alongside synthesis from carboxylic acids and esters in order to present a systematic study of the vapor-phase ketonization reaction realized under flow conditions in the presence of metal oxides (MnO_2_, CeO_2_ or ZrO_2_) deposited onto the surfaces of alumina or silica. The second part of our work is devoted to the synthesis of diacylbenzenes using ketonization. It has been known for more than a century that during the Friedel–Crafts acylation reaction of aromatic hydrocarbons, the introduction of the first acyl group to the hydrocarbon molecule makes it inactive for subsequent acylation. As a consequence, special methods for the synthesis of diacyl derivatives of aromatic hydrocarbons had to be developed. Our studies in this area have been focused on the single-step synthesis of the above-mentioned compounds under flow conditions in the presence of heterogeneous catalysts, which is in line with the principles of Green Chemistry.

## 2. Results

### 2.1. Characterization of Catalysts

#### 2.1.1. Powder X-ray Diffraction (PXRD)

**Figure 1:**
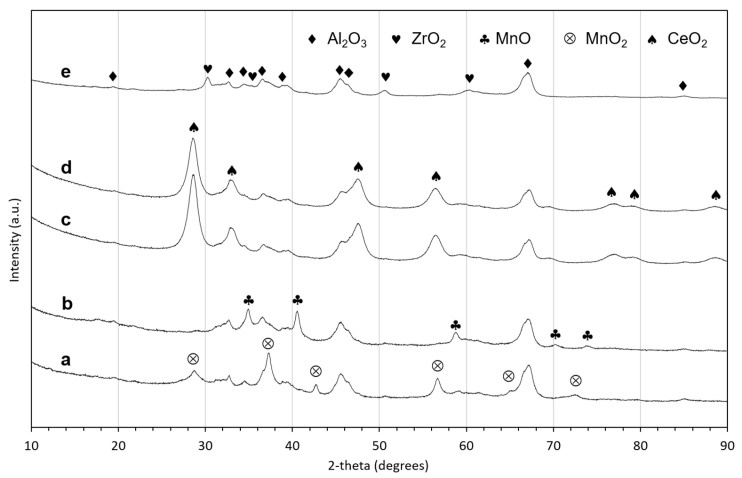
Diffraction patterns of the 20 wt.% MO_2_/Al_2_O_3_ catalysts (M = Mn, Ce or Zr); curve a—MnO_2_/Al_2_O_3_; curve b—MnO_2_/Al_2_O_3_ after reduction; curve c—CeO_2_/Al_2_O_3_; curve d—20 wt.% CeO_2_/Al_2_O_3_ after reduction; curve e—20 wt.% ZrO_2_/Al_2_O_3_.

The 20 wt.% MO_2_/Al_2_O_3_ catalysts were examined using the PXRD method (ex situ measurements). The main phases identified in the tested catalysts are indicated in Figure 1. In all of the samples, Al_2_O_3_ (PDF#46-1131) was identified. The diffraction patterns of the MO_2_/Al_2_O_3_ catalysts revealed that all of the active phases exhibited low or very low crystallinity due to the mild conditions of the transformations of catalyst precursors into catalysts (723 K, 3 h). Under these conditions, the sintering of the active phase as well as reactions between the active phase and the support are strongly inhibited. In the MnO_2_/Al_2_O_3_ sample before reduction, the main XRD reflection came from the Al_2_O_3_ support. The crystalline MnO_2_ (with a tetragonal structure, PDF#24-0753) was present, whereas in the sample after reduction, manganese(II) oxide—MnO (cubic structure, PDF#07-0230)—was noted. The average size of the MnO_2_ crystallites calculated using the (110) reflection (2θ = 28.8°, k = 1.0) was 11.5 nm. The average size of the MnO crystallites calculated using the (200) reflection (2θ = 40.5°, k = 1.0) was equal to 19.1 nm. In the case of the CeO_2_/Al_2_O_3_ catalyst, the diffraction patterns before and after reduction were practically the same, and regular CeO_2_ with a fluorite-type structure (PDF#81-0792) was identified with an average diameter of crystallites of approximately 7.6 ± 0.1 nm, respectively (calculated using the (111) reflection) (2θ = 28.5°, k = 1.0). The reason why, in this case, the main reflection from ceria is much more intense than that from alumina is that ceria gives higher counts than most oxides at the same concentration. The main reflection in the diffraction pattern of the 20 wt.% ZrO_2_/Al_2_O_3_ catalyst was again that of the support. It was noted that for both ZrO_2_ and MnO_2_ (Figure 1 curve a), the reflections from the active phase were not very prominent, which indicated a good dispersion of these oxides. In the powder diffraction pattern of ZrO_2_/Al_2_O_3_, the intensity of the active phase signals was too small to distinguish between tetragonal ZrO_2_ (PDF#800965) and cubic ZrO_2_ (PDF#27-0997), although the former is more likely to form under synthesis conditions.

#### 2.1.2. Scanning Electron Microscopy Coupled with Energy-Dispersive X-ray Spectroscopy (SEM-EDX)

**Figure 2:**
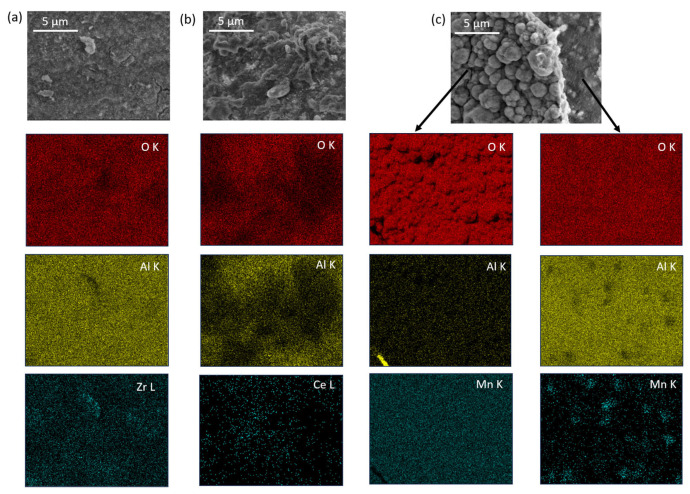
SEM-EDX results: SEM images and corresponding elemental maps of oxygen (red), aluminum (yellow) and the active component (blue) obtained for (**a**) 20 wt.% ZrO_2_/Al_2_O_3_, (**b**) 20 wt.% CeO_2_/Al_2_O_3_ and (**c**) 20 wt.% MnO_2_/Al_2_O_3_.

The topography and distribution of the active phase on the alumina support of the catalysts was studied with SEM-EDX. The SEM images and the elemental maps of aluminum, oxygen and Zr (Figure 2a), Ce (Figure 2b) and Mn (Figure 2c) are depicted in Figure 2. The surface of 20 wt.% ZrO_2_/Al_2_O_3_ shows a generally uniform distribution of oxygen, aluminum and zirconium. There was one small area where a slight enrichment of zirconium was accompanied by a slight depletion of aluminum. This is in line with the XRD results that show that these catalysts contain two phases: the alumina support and zirconia. The surface of the 20 wt.% CeO_2_/Al_2_O_3_ (Figure 2b) exhibits a slightly more complex topography than the zirconia/alumina species. The ceria is highly dispersed. In the case of the 20 wt.% MnO_2_/Al_2_O_3_ catalyst, two distinct types of surfaces are visible in Figure 2c: on the left side of the image, a nodular morphology can be seen, which appears to be a coating. From the elemental maps, it is clear that the coating was manganese oxide (EDX numbers indicate MnO_2_, which is consistent with the XRD analysis results) with a very low concentration of aluminum. In fact, the only area where an abundance of aluminum is detected is under the crack in the coating layer. It is noteworthy that the coating uniformly covers the alumina. Under the layer of manganese oxide, there is little manganese found on the alumina, and it is present as small patches of manganese accompanied by a local depletion of aluminum (Figure 2c).

#### 2.1.3. Temperature-Programmed Reduction (TPR)

**Figure 3:**
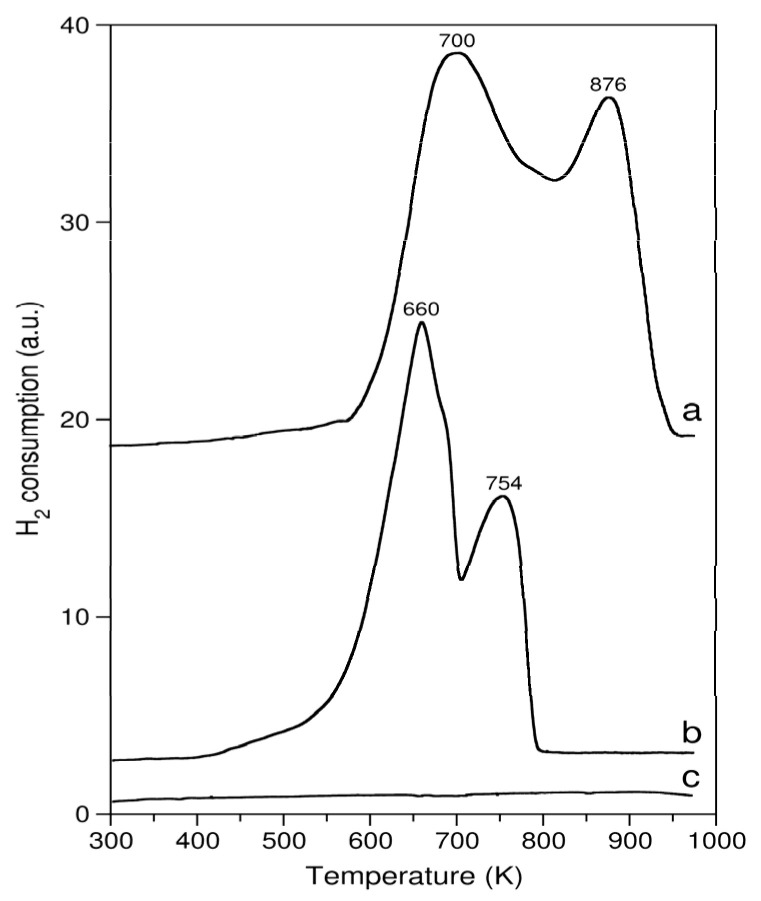
TPR profiles of curve a—pure MnO_2_; curve b—fresh 20 wt.% MnO_2_/Al_2_O_3_ catalyst; curve c—20 wt.% MnO_2_/Al_2_O_3_ catalyst after ketonization of propanoic anhydride (reproduced in part with permission from Elsevier).

**Figure 4:**
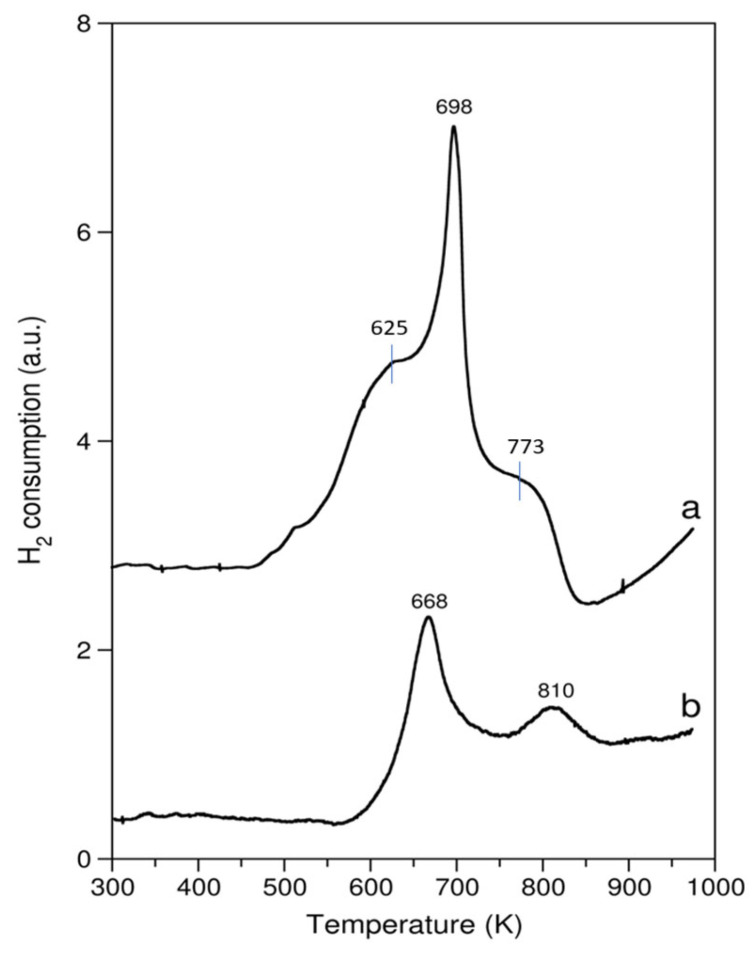
TPR profiles of the following: curve a—pure CeO_2_; curve b—20 wt.% CeO_2_/Al_2_O_3_ catalyst.

All of the 20 wt.% MO_2_/Al_2_O_3_ catalysts were characterized using TPR. Hydrogen uptake as a function of temperature is shown in Figure 3 and Figure 4, and the values of the hydrogen consumption are summarized in Table 1. In the case of pure ZrO_2_, only a residual consumption of hydrogen was observed at around 960 K. Therefore, the TPR measurement for the ZrO_2_/Al_2_O_3_ catalyst was not performed. The reduction of pure MnO_2_ started around 550 K. Two peaks indicating reduction were observed at 675 and 835 K. The consumption of hydrogen associated with the first peak corresponded to the formation of Mn_3_O_4_ (hausmanite). The second peak corresponded to the reduction of Mn_3_O_4_ to the lower manganese oxide—MnO (manganosite). MnO_2_ is not further reduced by hydrogen at temperatures below 1473 K. Based on the data obtained during the reduction of the MnO_2_/Al_2_O_3_ catalyst, it may be concluded that the hydrogen consumption profile is similar to that of pure MnO_2_ and the amount of hydrogen consumed corresponds to the reduction of MnO_2_ to MnO. The deposition of MnO_2_ onto the surface of Al_2_O_3_ has been noted to lead to lower temperatures of the maximum rate of hydrogen consumption compared to that obtained for the pure MnO_2_ phase.

The TPR profiles of the CeO_2_-containing catalysts were much more interesting (Figure 4). The reduction of pure CeO_2_ started at around 460 K, and four peaks of hydrogen consumption were detected at approximately 625, 696, 773 and above 940 K, of which the first three were not well resolved. The maximum hydrogen consumption rate occurred at 696 K. There were two peaks showing as shoulders before and after the main hydrogen consumption peak. These three hydrogen consumption peaks are most likely associated with the reduction of the surface oxygen of CeO_2_ [32]. The increasing hydrogen consumption above 940 K is attributed to the bulk reduction of CeO_2_ to Ce_2_O_3_ [32,33]. The reduction is not complete due to the termination of the ramp at 973 K. The negative peak for pure CeO_2_ around 850 K is suspected to originate from a release of CO, formed during the reduction of carbonates present on the surface of ceria [34], or a release of hydrogen accumulated on the surface of ceria during the TPR experiment [35].

By comparing the TPR results summarized in Table 1, it can be seen that MnO_2_ in either pure or supported form was quantitatively reduced to MnO by hydrogen. The same level of reduction of the active phase was observed when the catalyst was used in the ketonization of propanoic anhydride. The reduction of CeO_2_ and the cerium-containing catalyst by hydrogen within the temperature range of 300–973 K led to only a partial reduction of cerium dioxide. Moreover, the CeO_2_ active phase deposited onto the surface of the support showed a higher reducibility than pure CeO_2_ under the same reduction conditions. The active phase of the CeO_2_/Al_2_O_3_ catalyst was efficiently reduced by propanoic anhydride during its ketonization. This was indicated by the hydrogen consumption after the reaction. For pure ZrO_2_, only a residual consumption of hydrogen has been found.

Summing up this part of the study, pronounced differences between the reducibility of the three different catalysts can be seen. ZrO_2_ is a non-reducible oxide, whereas the other two exhibit substantially different temperature ranges and magnitudes of hydrogen consumption. Pure MnO_2_ starts to reduce at a lower temperature than pure ceria and is completely reduced to MnO by the end of the TPR program, as shown in Figure 3. In contrast, ceria is still undergoing a reduction at the final temperature of the measurement, and it does not reach the Ce_2_O_3_ stoichiometry. During the entire experiment, ceria consumes approx. 10 times less hydrogen than MnO_2_ (Table 1). It is noteworthy that the deposition of these oxides onto the alumina support drastically changes the reducibility of both MnO_2_ and CeO_2_. In the case of MnO_2_, alumina impacts the overall hydrogen consumption to a smaller extent than CeO_2_, but it shifts the reduction to lower temperatures. The MnO_2_ supported on alumina is fully reduced to MnO at a temperature 150 degrees lower than the unsupported MnO_2_. The reverse is noted for ceria, i.e., the deposition of ceria onto alumina leads to a higher reduction onset temperature. Nevertheless, the overall hydrogen consumption of hydrogen by 20 wt.% CeO_2_/Al_2_O_3_ is higher than that of pure ceria (Table 1).

#### 2.1.4. Nitrogen Physisorption

The values of the specific surface area of all of the studied catalysts were lower than those measured for the pure supports irrespective of their types (Table 2). The spent catalysts exhibited lower specific surface areas than the fresh ones. In addition, the ketonization of diisopropyl terephthalate with acetic acid on manganese and cerium catalysts led to a larger decrease in the specific surface area than the ketonization of propionic anhydride. This was probably caused by a higher degree of coke formation on the surface of the catalyst in the case of reactants which possess an aromatic ring in their structures.

### 2.2. Activity Measurements

#### 2.2.1. Homo- and Cross-Ketonization of Aliphatic Anhydrides

A series of six aliphatic anhydrides of monocarboxylic acids were studied in the vapor-phase ketonization reaction under flow conditions in the presence of 20 wt.% MO_2_/S (ex-nitrate) catalysts ((M = Mn, Ce or Zr; S = SiO_2_ or Al_2_O_3_). The homo-ketonization of anhydrides occurs via Equation (7):

(RCO)_2_O → R_2_C=O + CO_2_ (7)

When two different anhydrides react, the reaction is called cross-ketonization. Three ketones are shown as reaction products in Equation (8):

(RCO)_2_O + (R’CO)_2_O → R_2_C=O + RR’C=O + R’_2_C=O + 2CO_2_ (8)

First, propanoic anhydride was transformed into 3-pentanone using 20 wt.% MO_2_/S, where S = SiO_2_ or Al_2_O_3_ and M = Ce or Mn (Table 3). These results are consistent with the stoichiometry shown in Equation (9). In the preliminary studies, the catalytic activity levels of the grains of fused quartz, quartz wool and the quartz walls of the reactor were measured. It was found that at 623 K, the background activity level was only 3% conversion. Next, the activity levels of the pure supports, SiO_2_ or Al_2_O_3_, in the reaction were determined. Pura silica showed very moderate activity. At 623 K, only 8% conversion was noted. Pure alumina was much more active at the same temperature, at which a 39% conversion of anhydride was found.

(C_2_H_5_CO)_2_O → (C_2_H_5_)_2_C=O + CO_2_ (9)

**Figure 5:**
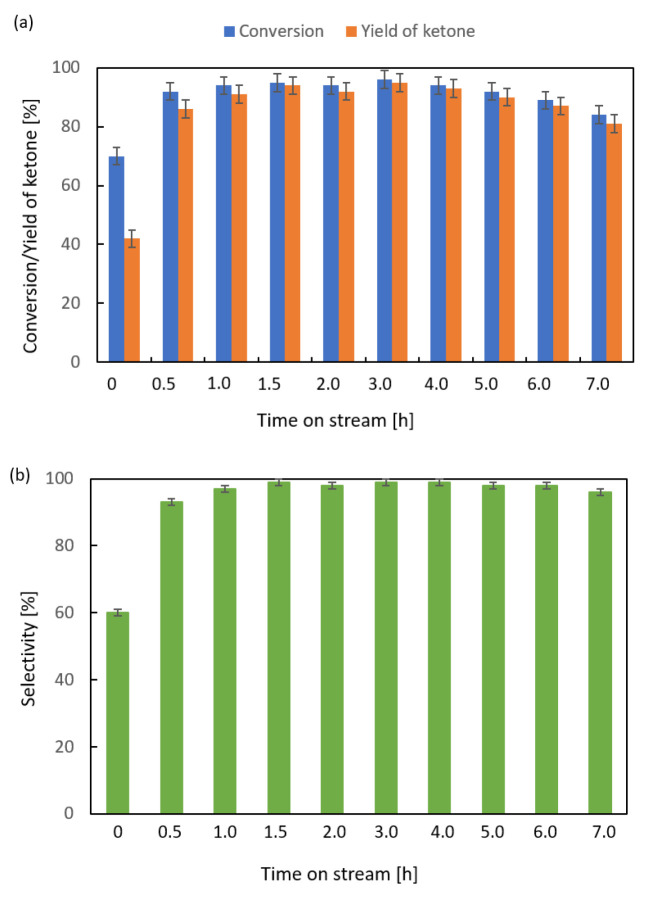
Time-on-stream activity of 20 wt.% MnO_2_/Al_2_O_3_ catalyst in ketonization of propanoic anhydride at 598 K; LHSV = 3 cm^3^ g^−1^ h^−1^, V_N2_ = 3 dm^3^·h^−1^; (**a**) conversion and yield of ketones and (**b**) selectivity.

Both supported CeO_2_ catalysts were more active than their respective supports. The deposition of CeO_2_ onto the surface of alumina led to higher activity than that observed for the CeO_2_/SiO_2_ catalyst. The same behavior was observed for the two manganese catalysts. The highest activity in the ketonization of propanoic anhydride was observed for the MnO_2_/Al_2_O_3_ catalyst. That catalyst caused a 99% conversion of the anhydride and a 97% yield of 3-pentanone at 623 K. Therefore, a time-on-stream activity test was performed with this catalyst at 598 K (Figure 5). In the initial 1 h, the conversion of anhydride increased from 70 to 94%, and the yield of ketones increased from 42 to 91%. In the next 3 h, the conversion stabilized at 94–96% before slowly decreasing. The decrease was caused by partial coking of the catalyst. After 7 h of continuous operation, the catalyst was still very active, with a conversion of 84% and an 81% yield of 3-pentanone.

The Ce and Mn catalysts deposited on alumina were chosen for further studies of ketonization of ethanoic, *n*-butanoic and *n*-heptanoic anhydrides. The results are summarized in Table 4. The reactivity of anhydrides using pure Al_2_O_3_ increases as the molecular weight of the anhydride increases. At a temperature of 673 K, an increasing series of anhydride conversion values was observed: ethanoic (11%) < butanoic (27%) < heptanoic (70%). Catalysts containing MnO_2_ as the active phase were more active in the reaction than their cerium counterparts, regardless of the type of anhydride. The highest yields of acetone, 4-heptanone and 7-tridecanone, which were 87%, 91% and 97%, respectively, were obtained within the temperature range of 648–698 K. In the literature [36], a study involving the formation of 4-heptanone from *n*-butanoic acid at 723 K using Al_2_O_3_ yielded 78% of ketones. The temperature of the study was only slightly higher than the highest temperature used in our study (by 25 degrees), but the significantly higher yield is understandable in the context of the much lower weight hourly space velocity.

The next stage of research was devoted to the catalytic transformations of branched anhydrides. For this purpose, reactions involving a single branched anhydride, known as the 2-methylpropanoic (isobutyric) anhydride, and a doubly branched anhydride, known as the 2,2-dimethylpropanoic (pivalic) anhydride, were carried out, and the results are summarized in Table 5. For both anhydrides, an increasing conversion with the reaction temperature was observed. The only ketone formed was 2,4-dimethyl-3-pentanone. The reactivity of the 2-methylpropanoic anhydride was much lower compared to the reactivity of the straight-chained anhydrides. Yields of ketones exceeding 50% were reached at temperatures above 673 K. The highest yield of ketones was 80% and was found at the highest temperature (723 K). The pivalic anhydride underwent transformations, which did not lead to the formation of 2,2,4,4-tetramethyl-3-pentanone (pivalone); instead of ketones, light hydrocarbons were found among the reaction products. Based on the negative results using a pivalic anhydride in the synthesis of pivalone, as well as from previous attempts to use pivalic acid and its ethyl ester (unpublished results), it seems obvious that this ketone cannot be obtained in the ketonization reaction.

Summarizing this part of the study, it can be stated that in the case of straight-chain anhydrides (ethanoic, propanoic, butanoic and heptanoic), high conversions (above 80%) were accompanied by equally high yields of corresponding ketones. The selectivity of the reaction to ketones usually exceeded 90%. Other reaction products were light hydrocarbons (decomposition products of anhydrides). In the case of a branched anhydride, such as the 2-methylpropanoic anhydride, its reactivity was significantly lower when compared to the straight-chain anhydrides. A high selectivity to ketones, 88 to 96%, was noted for both tested catalysts. However, in the case of the 2,2-dimethylpropanoic anhydride, the selectivity to ketones was 0% over the entire tested temperature range. The only reaction products were the decomposition products of the anhydride, i.e., *i*-butene and carbon oxides.

Attempts were made to perform the cross-ketonization of an equimolar mixture of propanoic and *n*-heptanoic anhydrides in the presence of both cerium and manganese catalysts. The reaction products were three ketones: 3-pentanone, 3-nonanone and 7-tridecanone. The compositions of the post-reaction mixtures obtained at a temperature of 698 K are reported in Table 6. The two anhydrides were found to be able to react with each other in the cross-ketonization reaction and form 3-nonanone as the main product. Yields as high as 68% and 59% were noted for the cerium and manganese catalysts, respectively. The main product was accompanied by two symmetrical ketones: a light one, 3-pentanone, and a heavy one, namely 7-tridecanone. Their yields ranged from 14 to 20%.

#### 2.2.2. Cross-Ketonization of Dialkyl Benzenedicarboxylates with Acetic Acid

Benzenedicarboxylic acids occur as high-melting solids, which makes their dosing into the flow reactor difficult. Convenient precursors for these acids are their dialkyl esters, which are mostly liquids, and as liquids or as solutions in acetic acid, they can be conveniently dosed into the reactor. The course of the cross-ketonization reaction of selected alkyl esters of benzene-1,x-dicacarboxylic acids for x = 2, 3 or 4 with acetic acid was examined for the synthesis of the appropriate 1,x-diacetylbenzenes, as shown in Table 7, Table 8, Table 9 and Table 10.

Attempts to synthesize 1,2-diacetylbenzene through the ketonization of acetic acid with dialkyl (ethyl and isopropyl) phthalates were unsuccessful. Within the temperature range of 673–723 K, using 20 wt.% MO_2_/Al_2_O_3_ (M = Ce or Mn) catalysts, acetophenone was obtained as the main reaction product with yields up to 40%. The absence of 1,2-diacetylbenzene in the reaction products was noted. Diisopropyl isophthalate was used as the precursor of benzene-1,3-dicarboxylic acid. Its reaction with acetic acid, carried out in the presence of the 20 wt.% MO_2_/Al_2_O_3_ catalysts, was the subject of our research (Table 7). Two main reaction pathways were observed. The first one was the cross-ketonization of the ester with acetic acid, resulting in the formation of 1,3-diacetylbenzene, as shown in Equation (10).



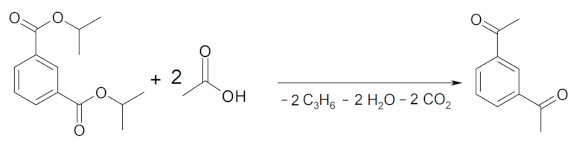

(10)


The second path describes the formation of acetophenone—the main by-product of the reaction presented in Equations (11) and (12). Very low yields of 1,3-diacetylbenzene (1,3-DAB) (2–8%) were formed for all 20 wt.% MO_2_/Al_2_O_3_ (M = Ce, Mn or Zr) catalysts. The highest yield (8%) was obtained at the highest temperature (723 K) for the manganese and cerium catalysts. The yield of acetophenone always exceeded the yield of 1,3-diacetylbenzene at the same temperature. The ZrO_2_-containing catalyst was less active; in its presence, only a 3% yield of 1,3-DAB was observed. Attempts to increase the activity of the cerium catalyst by changing the type of precursor used for its synthesis were ineffective. The catalysts obtained using either CeCl_3_·7H_2_O or (NH_4_)_2_Ce(NO_3_)_6_ as CeO_2_ precursors were less active in the reaction.



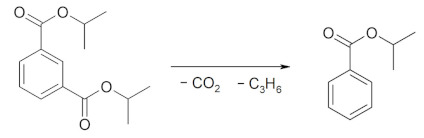

(11)




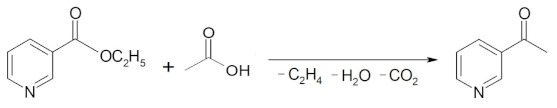

(12)


In part of the research devoted to the cross-ketonization reaction of acetic acid with dialkyl terephthalates to synthesize 1,4-diacetylbenzene (1,4-DAB), the diisopropyl ester of terephthalic acid was used as the starting precursor of the acid for the results shown in Table 8. All 20 wt.% MO_2_/Al_2_O_3_ (M = Ce, Mn or Zr) catalysts were tested in the reaction. At 698 K, the highest yield of 1,4-DAB was observed for all catalysts. The CeO_2_/Al_2_O_3_ catalyst definitively showed the highest activity. Using the CeO_2_/Al_2_O_3_ catalyst, the 1,4-DAB yields were in the range of 31–35% for temperatures of 698 and 723 K. An attempt to increase the activity of this catalyst by changing the precursor of the active phase to (NH_4_)_2_Ce(NO_3_)_6_ was not successful, as shown in Table 8. It was noted that acetophenone was the dominant product of the reaction in the presence of the MnO_2_/Al_2_O_3_ catalyst.

In the next step, the cross-ketonization reaction of acetic acid with other alkyl terephthalates was tested using pure alumina and the 20 wt.% CeO_2_/Al_2_O_3_ catalyst. The following alkyl terephthalates were used: dimethyl, diethyl, diisopropyl and monoisopropyl. The results are reported in Table 9. Pure alumina only showed residual activity in the reaction. Using the CeO_2_/Al_2_O_3_ catalyst, a decreasing set of ester reactivities was noted (the yield of 1,4-DAB in per cent was given in brackets): diisopropyl (35) > diethyl (23) > dimethyl (6). Monoisopropyl terephthalate was not able to be tested because when it is mixed with acetic acid, a solid product is formed. Acetophenone was always found among the reaction products with yields ranging from 6 to 38% depending on the type of ester and reaction temperature. Time-on-stream tests were performed at a temperature of 698 K with two dialkyl terepthalates. The results are presented in Table 10. For the time range of 2–4 h, both reactions led to a maximum yield of 1,4-DAB of approx. 40–42% (Table 10). The yield of acetophenone was roughly half that of 1,4-DAB in this reaction in the same time range. In the fifth hour of the reaction, slight decreases in the yields of 1,4-DAB (37%) and acetophenone (16%) were noted due to the coking of the catalyst. The yields of acetophenone were slightly lower in the case of the reaction with diethyl terephthalate.

#### 2.2.3. Cross-Ketonization of Substituted Alkanoic Acids with Acetic Acid

In the third and last part of the work, the usefulness of the cross-ketonization reaction with ω-substituted carboxylic acids containing aromatic or heterocyclic substituents, with O, S or N atoms in the ring, was examined. In the initial phase of the cross-ketonization studies, ω-phenyl alkanoic acids and acetic acid were reacted using cerium and manganese catalysts (Table 11). Irrespective of the type of catalyst used, yields of ketones as high as 47–49% for 3-phenylpropanoic, 4-phenylbutanoic and 3-(4-methoxyphenyl)propanoic acids were obtained. In the literature [37], yields of ketones using MnO_2_ catalysts were reported to be as high as 95% for long straight-chain carboxylic acids at temperatures of approx. 620 K. These are much higher than those observed in our study. The difference might be caused by the fact that in the case of homo-ketonization, only one ketone was formed instead of three. A comparison of the respective ketone yields due to different synthesis methods has been reported by us previously [15]. The presence of a vinyl group in cinnamic acid diminished its reactivity in the studied reaction. In this case, the yield of the corresponding ketone did not exceed 20%.

The next step was to study the cross-ketonization reaction of acetic acid with acids containing furan, thiophene or piperidine rings as substituents (Table 12). 2-Thiopheneacetic acid underwent a ketonization reaction with very moderate yields of 2-thiopheneacetone that did not exceed 14%. The acid underwent homo-ketonization, following the reaction shown in Equation (13), with the formation of 1,3-bis(2,2′-dithienyl)-2-propanone, although with a yield below 5%.



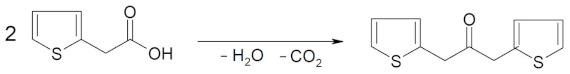

(13)


Using 3-(2-furyl)acrylic acid, only residual yields of the appropriate ketone (2–3%) were obtained. An attempt to mix acetic acid with 4-piperidinecarboxylic acid downstream of the synthesis reactor led to their reaction at room temperature, resulting in a highly viscous liquid product that was no longer dosed into the reactor. The next group of compounds intended for cross-ketonization studies were alkyl esters of carboxylic acids containing pyridyl or phenyl substituents in the ω position of an ester molecule. In the case of the cross-ketonization of acetic acid with ethyl nicotinate, the reaction occurs via Equation (14):



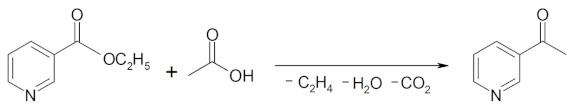

(14)


The results of the cross-ketonization reaction of acetic acid with ethyl nicotinate carried out using 20 wt.% CeO_2_/Al_2_O_3_, 20 wt.% MnO_2_/Al_2_O_3_ catalysts and pure Al_2_O_3_ are summarized in Table 13. The yield of 3-acetylpyridine for all catalysts did not exceed 7%. Pure Al_2_O_3_ was slightly more active than the manganese catalyst. A time-on-stream test performed at 698 K revealed that the CeO_2_/Al_2_O_3_ catalyst remained unchanged during the 4-h residence time with a yield of 3-acetylpridine at 5–7%.

The last topic of our research was to determine the reactivity of alkyl esters of carboxylic acids substituted with aryl groups (phenyl and naphthyl) in the cross-ketonization reaction with acetic acid. The results are shown in Table 14. The results clearly indicate that the esters of the acids that were tested are compounds of moderate reactivity and that the course of the reaction strongly depends on the structure of the ester. In the case of the cross-ketonization of ethyl 4-nitrophenylacetate with acetic acid, within the tested temperature range, no formation of the corresponding ketone was recorded; only products of deep ester decomposition were formed: 4-toluidine and 4-nitrotoluene. At 723 K, ethyl 2-naphthoate was shown to undergo cross-ketonization with acetic acid with the formation of 2-naphthyl methyl ketone with a yield as high as 41%. As in the case of cinnamic acid, its ethyl ester showed diminished reactivity in the reaction. The yield of the corresponding ketone was 13% and decreased with an increasing temperature due to the side reaction leading to styrene.

## 3. Materials and Methods

### 3.1. Catalysts

Commercial silica (Aerosil 200, Degussa, Frankfurt, Germany) and alumina (type C, Degussa, Frankfurt, Germany) were used as supports. A powder of each oxide was mixed with redistilled water, and the resulting gel was dried at 333 K and 393 K for 24 h. The sieved fraction with a 0.4–0.6 mm grain diameter (0.63–1.25 mm for the ketonization of esters of 1,x-benzenedicarboxylic acids) was calcined at 873 K for 6 h in a stream of air. The 20 wt.% MO_2_/SiO_2_ or 20 wt% MO_2_/Al_2_O_3_ catalysts, where M = Mn, Ce or Zr, were prepared by the impregnation of the grains of support using the incipient wetness technique. Mn(NO_3_)_2_·4H_2_O (purum p.a., Fluka, Buchs, Switzerland), Ce(NO_3_)_3_∙6H_2_O (p.a. > 99.0%, Aldrich, Saint Louis, MO, USA) (NH_4_)_2_Ce(NO_3_)_6_ (99%, Aldrich), CeCl_3_·7H_2_O (purum p.a., >98%, Aldrich, Saint Louis, MO, USA), ZrO(NO_3_)_2·_2H_2_O (1% Hf content, Biddle, Sawyer Co., London, UK) and ZrOCl_2_·8H_2_O (Aldrich, 98%) were used as the precursors of active phases. The impregnated samples were dried at 393 K for 12 h and calcined at 723 K for 3 h in a stream of air. The reduced catalysts were obtained by heating the 2 g samples at 723 K for 3 h in a stream of hydrogen (3 dm^3^·h^−1^). The surface area of the support and catalysts was measured using a Micromeritics (Norcross, GA, USA) ASAP 2020 instrument. Pure CeO_2_ was obtained through the decomposition of solid (NH_4_)_2_Ce(NO_3_)_6_ (99%, Aldrich, Saint Louis, MO, USA), first at 473 K in stream of air for 1 h and then at 553 K for 3 h. Pure ZrO_2_ was prepared through the decomposition of ZrO_2_·nH_2_O in a muffle oven at 873 K for 3 h in air. Synthesis of pure MnO_2_ was described elsewhere [14].

### 3.2. Reagents

Propionic anhydride (pure, BDH, London, UK) was purified by double fractional distillation under normal pressure, b. p. 440–441 K, with a purity of 99.7% (acidimetric), n^20^_D_ = 1.4039 (exp.). A sequence of aliphatic anhydrides purchased from Aldrich were used (Saint Louis, MO, USA): acetic (purity 99%), butyric (98%), isobutyric (97%), pivalic (99%) and heptanoic (96%) anhydrides. They were distilled under a normal/diminished pressure, and the middle fractions obtained were stored in tight containers to avoid hydrolysis.

Terephthaloyl chloride (99%, Aldrich, Saint Louis, MO, USA), isophthaloyl chloride (99%, Aldrich, Saint Louis, MO, USA), terephthalic acid (98%, Aldrich, Saint Louis, MO, USA) and phthalic anhydride (>99%, Aldrich, Saint Louis, MO, USA) were used as starting materials for the synthesis of alkyl benzenedicarboxylates. Methyl terephthalate was prepared in 80% yield in the reaction of terephthalic acid (0.2 mol) with trimethyl orthoformate (99%, Aldrich, Saint Louis, MO, USA) (1 mol) under reflux for 8 h. Ethyl and isopropyl terephthalates were prepared by adding terephthaloyl chloride in small portions to the excess of anhydrous alcohols heated to 50 °C. The resulting solutions were heated to reflux for 10 h. After cooling, the volatiles were evaporated under reduced pressure (rotary evaporator), and the residues were dissolved in toluene. The obtained solutions were carefully washed with a 5% solution of NaHCO_3_ and then with water, dried over MgSO_4_ and evaporated to dryness. Final purification was achieved by fractional distillation of the esters under reduced pressure. Ethyl and isopropyl terephthalates were obtained with yields of 92 and 90%, respectively.

Isopropyl hydrogen terephthalate was synthesized by adding KOH (0.1 mol) into the solution of diisopropyl terephthalate (0.1 mol) in 300 cm^3^ of isopropyl alcohol and heating the mixture under reflux for 4 h. After cooling, the solvent was distilled and the residue was treated with 250 cm^3^ of water. The product, which is insoluble in water, was extracted with CH_2_Cl_2_. The water solution was acidified with a concentrated HCl aqueous solution, and the resulting precipitate was extracted with diethyl ether. The obtained extract was dried over MgSO_4_ for 24 h. The volume of the resulting anhydrous solution was decreased under reduced pressure using a rotary evaporator, and it provided the colorless, transparent ester as a solid (48% yield). A series of carboxylic acids were used as received: 2-thiopheneacetic (98%, Aldrich, Saint Louis, MO, USA), 3-(2-furyl)acrylic (99%, Aldrich, Saint Louis, MO, USA), 4-piperidinecarboxylic (97%, Aldrich, Saint Louis, MO, USA), 3-phenylpropionic (99%, Aldrich, Saint Louis, MO, USA), 4-phenylbutyric (99%, Aldrich, Saint Louis, MO, USA) 3-(4-methoxyphenyl)propionic (99%, Aldrich, Saint Louis, MO, USA), nicotinic (pure, Loba-Chemie, Vienna, Austria), 2-naphthoic (98%, Aldrich, Saint Louis, MO, USA) and cinnamic (98%, Biddle, Sawyer & Co., Ltd., London, UK) acids. Ethyl esters: cinnamate, 2-(4-nitrophenyl)acetate and 2-naphthoate were prepared through direct esterification of the corresponding acids with anhydrous ethanol in the presence of 98% H_2_SO_4_ as a catalyst. Crude products were concentrated in a rotary evaporator, washed with 5% solution of NaHCO_3_, dried over MgSO_4_ and finally separated by fractional distillation under reduced pressure. Ethyl nicotinate was prepared by mixing nicotinic acid (0.3 mol) with 180 cm^3^ of ethanol and slowly adding concentrated H_2_SO_4_ (50 cm^3^). The mixture was heated with reflux for 4 h. The post-reaction mixture was diluted with 200 cm^3^ of cold water and alkalized with ammonia solution (400 cm^3^). The organic layer was separated, and the water layer was extracted with CH_2_Cl_2_ (2 × 20 cm^3^). Organic layers were collected, washed with water and dried over MgSO_4_. After evaporating the solvent, the crude ester was distilled under reduced pressure. The resultant yield was 56%. Purity of esters exceeded 98.5–99.0%, as determined by GC.

### 3.3. Ketonization of Anhydrides, Acids and Esters

The reactions were carried out in a typical quartz flow reactor with a fixed catalyst bed with 1.000 g of catalyst within the temperature range of 523–723 K in a stream of nitrogen (flow 50 cm^3^·min^−1^). The hourly liquid space velocity (HLSV) of reactants was equal to 3 cm^3^ g^−1^ h^−1^. Post-reaction mixtures were passed through a water condenser and collected in a glass container chilled in dry ice—2-propanol bath. Reaction products collected during the first 60 min of the reaction were discarded, and the analytical samples of the reaction products were taken during next 30 min in the reaction stream. Due to the fact that the concentration of the appropriate carboxylic acid formed as a side product in the studied reactions did not exceed 0.3% for all samples taken for analysis, its concentration was omitted in the results. Samples were analyzed using a chromatograph (HP-6890, Agilent, Santa Clara, CA, USA) equipped with HP-1 column (length 60 m, 0.25 i.d.), FID. The quantitative analysis was performed using *n*-decane, *n*-dodecane or *n*-tetradecane as internal standards. The compounds were identified by GC-MS (HP-6890N, Agilent, Santa Clara, CA, USA) with a 5973N (Agilent, Santa Clara, CA, USA) mass detector. Independently, the content of anhydride in post-reaction mixtures was determined by titration with standard 0.1 M KOH solution in the presence of phenolphthalein as the indicator. The end point of titration was taken after 1 min without the discoloration of the solution, which ensured the quantitative conversion of a given anhydride, caused by a slow rate of its hydrolysis, into acid.

### 3.4. Ketonization of Dialkyl Benzenedicarboxylates

The reactions were carried out in a quartz flow reactor (length 300 mm, i.d. 15 mm) filled with 5.00 g of catalyst and 3.0 g of fused quartz (0.8–1.25 mm) placed on top of the catalyst bed. The measurements were performed within the temperature range of 673–723 K in a stream of nitrogen (flow 50 cm^3^·min^−1^) with the hourly liquid space velocity (HLSV) of reactants of 2 cm^3^ g^−1^ h^−1^. The reaction products formed during the first 60 min of the reaction were discarded, and the analytical samples of the reaction products were taken during the next 60 min in the reaction stream for every temperature studied (673, 698 and 723 K). Due to the fact that the benzenedicarboxylic acids as well as diacetylbenzenes are high-melting solids, the post-reaction mixtures were collected by passing vapors of the products through a known volume of acetone (10 cm^3^) and washing out the solid deposit condensed above on the walls of the condensation tube with an additional volume of acetone. The solutions in acetone were concentrated under a reduced pressure (rotary evaporator), and the obtained residue was treated with 10 cm^3^ of CH_2_Cl_2_. The extract was washed with 2% solution of NaOH until a neutral pH was obtained, and then with water, and dried over MgSO_4_. Diphenylmethane, used as internal standard, was added to the extract, and a whole sample was dissolved in CH_2_Cl_2_. Samples were analyzed using a gas chromatograph (HRGC 4000B, KONIK, Barcelona, Spain) equipped with TR WAX column (length 30 m, 0.25 mm i.d., film thickness 0.25 μm), FID.

### 3.5. XRD Analysis

Powder X-ray diffraction patterns were recorded at room temperature on a Bruker D8 Advance diffractometer (Bruker AXS, Karlsruhe, Germany) equipped with a LYNXEYE position-sensitive detector using Cu-Kα radiation (λ = 0.15418 nm). The data were collected in Bragg–Brentano (θ/θ) horizontal geometry (flat reflection mode) between 8° and 90° (2θ) in a continuous scan, using 0.03° steps with 2 s/step (total time 384 s/step). Data were collected under standard laboratory conditions.

### 3.6. Scanning Electron Microscopy Coupled with Energy-Dispersive X-ray Spectroscopy (SEM-EDX)

The microscope used for the imaging and composition determination was the PFIB Helios 5 DualBeam instrument (Thermo Fisher Scientific, Dreieich, Germany). Images of the surface were obtained in the electron beam mode with an Everhart–Thornley secondary electron detector with a voltage of 5 kV, a beam current of 13 pA and a working distance of 3.7 mm at different magnifications. A magnification of 10,000 times was used to acquire the elemental maps for the O K, Al K, Mn K, Ce L and Zr L lines with parameters suitable to obtain the following appropriate intensity counts: beam voltage = 10 kV, beam current = 0.4 nA, dwell time = 200 µs, resolution = 256 × 200 and 20 frames.

### 3.7. Nitrogen Physisorption

The total specific surface area of the catalysts was determined by nitrogen physisorption using the ASAP2020 device (Micromeritics Instrument Co., Norcross, GA, USA). Before the measurement, the samples were outgassed for 2 h at 150 °C, and nitrogen adsorption was carried out at the temperature of liquid nitrogen. The total specific surface area (S_BET_) was determined using the BET (Brunauer–Emmett–Teller) adsorption isotherm model in the relative pressure range (p/p_0_) of 0.05–0.3.

### 3.8. Temperature-Programmed Reduction (TPR)

Temperature-Programmed Reduction was carried out in a flow system with a linearly increasing temperature. A sample of a catalyst was placed in a quartz reactor heated using an electric furnace. A mixture of hydrogen in argon (6% *v*/*v*) was used as the reductant. The TPR profiles were recorded in the range of 323–973 K. The temperature of the sample was measured using a thermocouple and controlled by an Omega 2010 temperature controller. Changes in the hydrogen concentration were monitored using a Gow-Mac thermal conductivity cell. Analog signals from the thermocouple and TCD were passed through digital voltmeters and monitored using an 8255 I/0 card in a microcomputer.

## 4. Conclusions

The ketonization of anhydrides, acids and esters was performed using a flow system in the presence of 20 wt.% MO_2_/Al_2_O_3_ (M = Ce, Mn, Zr). In general, the cerium and manganese oxides showed superior activity to the zirconium oxide catalysts. The support exhibited much lower activity in the studied reactions than the 20 wt.% MO_2_/Al_2_O_3_ systems. Six anhydrides were tested, and the reactivity was found to increase with the molecular weight of the anhydride. It was shown that anhydrides of aliphatic monocarboxylic acids undergo catalytic homo-ketonization to the corresponding ketones with high yields (85–95%). The presence of a branched substituent in the 2-methylpropanoic anhydride molecule causes its reduced reactivity. Despite this, at 723 K, 2,4-dimethyl-3-pentanone was obtained with a maximum yield of 80%. There was no formation of the corresponding ketone in the case of the 2,2-dimethylpropanoic anhydride because the substrate decomposed into low-molecular-weight products under the reaction conditions. Moreover, it has been shown that the cross-ketonization of a mixture of the two different anhydrides results in the synthesis of a non-symmetric ketone with yields of up to 60–70%. The studies of the ketonization of dialkyl benzenedicaboxylates with acetic acid have shown that the yield of 1,x-diacetylbenzenes increases in the following order: x = 4 > x = 3 > x = 2. Yields as high as approx. 40% of appropriate 1,4-diacetylbenzes were obtained in this study, with 1,3-diacetylbenzenes being present in concentrations below 8%. No 1,2-diactetylbenzene was obtained. In the last part of our work, we demonstrated the usefulness and possible limitations of the ketonization reaction depending on the structures of carboxylic acids and their esters. The presence of heterocycle substituents in the structures of acids dramatically changes their properties. For instance, nitrogen atom(s) in such a substituent causes the appearance of basic properties of this part of a molecule, which exhibits a detrimental effect on the reactivity of an acid. A substituent with oxygen and sulfur were also used, and both decreased the yield of the corresponding ketones, but to a smaller extent than the nitrogen-containing substituents.

## Figures and Tables

**Table 1:** The results of TPR measurements of 20 wt.% MO_2_/Al_2_O_3_ catalysts (M = Mn, Ce or Zr).

Catalyst	H_2_/MO_2_ Molar Ratio in TPR
MnO_2_ ^1^	0.98
MnO_2_/Al_2_O_3_	1.04
MnO_2_/Al_2_O_3_ ^2^	0.03
CeO_2_ ^3^	0.11
CeO_2_/Al_2_O_3_	0.26
CeO_2_/Al_2_O_3_ ^2^	0.03
ZrO_2_ ^4^	~0.0

^1^ Pure MnO_2_; ^2^ spent catalyst; ^3^ pure CeO_2_; ^4^ pure ZrO_2_.

**Table 2:** Specific surface area of fresh and spent 20 wt.% MO_2_/S (ex-nitrate) catalysts (M = Mn, Ce or Zr; S = SiO_2_ or Al_2_O_3_).

Catalyst	S_BET_ (m^2^ g^−1^)	S_BET_ ^1^ (m^2^ g^−1^)
-- ^2^	208	--
CeO_2_/SiO_2_	188	169 ^3^
MnO_2_/SiO_2_	179	156 ^3^
-- ^4^	103	--
MnO_2_/Al_2_O_3_	82	63 ^3^
		60 ^5^
CeO_2_/Al_2_O_3_	85	67 ^3^
		62 ^5^
ZrO_2_/Al_2_O_3_	95	68 ^5^

^1^ Spent catalyst; ^2^ pure SiO_2_ support; ^3^ after ketonization of propionic anhydride; ^4^ pure Al_2_O_3_ support; ^5^ after ketonization of diisopropyl terephthalate with acetic acid.

**Table 3:** Activity of 20 wt.% MO_2_/S catalysts (M = Mn or Ce; S = SiO_2_ or Al_2_O_3_) in ketonization of propanoic anhydride; LHSV = 3 cm^3^ g^−1^ h^−1^, V_N2_ = 3 dm^3^ h^−1^.

Catalyst	Conversion/Yields of Ketones (%)/(%)				
	573 K	598 K	623 K	648 K	673 K
-- ^1^	0/-	1/0	3/1	6/2	11/4
-- ^2^	1/0	5/3	8/5	22/17	32/27
CeO_2_/SiO_2_	30/27	47/45	70/67	84/80	89/81
MnO_2_/SiO_2_	68/66	90/88	96/94	98/95	--
-- ^3^	18/16	27/25	39/33	54/49	--
CeO_2_/Al_2_O_3_	52/50	77/75	91/87	99/94	--
MnO_2_/Al_2_O_3_	86/84	97/95	99/97	99/95	--

^1^ The activity of 1.00 g of fused quartz grains, quartz wool and the quartz walls of the reactor; ^2^ pure SiO_2_; ^3^ pure Al_2_O_3_.

**Table 4:** Activity of 20 wt.% MO_2_/Al_2_O_3_ (ex-nitrate) catalysts (M = Mn or Ce) in ketonization of ethanoic, *n*-butanoic and *n*-heptanoic anhydrides; LHSV = 3 cm^3^ g^−1^ h^−1^, V_N2_ = 3 dm^3^ h^−1^.

Anhydride	Active Phase	Conversion/Yields of Ketones (%)/(%)				
		598 K	623 K	648 K	673 K	698 K
(CH_3_CO)_2_O	-- ^1^	--	3/0	8/0	11/1	16/4
	CeO_2_	32/27	84/82	95/79	96/68	--
	MnO_2_	33/22	87/84	95/87	98/84	--
(*n*-C_3_H_7_CO)_2_O	-- ^1^	--	6/2	12/5	27/18	37/26
	CeO_2_	6/1	13/6	44/35	78/71	93/84
	MnO_2_	36/30	73/69	89/82	93/85	97/91
(*n*-C_6_H_13_CO)_2_O	-- ^1^	41/38	57/52	63/59	70/62	78/52
	CeO_2_	68/65	82/78	93/88	96/90	98/91
	MnO_2_	90/89	98/96	99/97	99/96	99/95

^1^ Pure alumina.

**Table 5:** Activity of 20 wt.% MO_2_/Al_2_O_3_ catalysts (M = Mn or Ce) in ketonization of 2-methylpropanoic and 2,2-dimethylpropanoic anhydrides; LHSV = 3 cm^3^ g^−1^ h^−1^, V_N2_ = 3 dm^3^ h^−1^.

Anhydride	Active Phase	Conversion/Yields of Ketones (%)/(%)				
		623 K	648 K	673 K	698 K	723 K
((CH_3_)_2_CHCO)_2_O	-- ^1^	1/0	2/0	4/0	6/0	15/0
	CeO_2_	5/1	8/5	22/16	40/37	57/55
	MnO_2_	5/1	9/4	11/6	60/53	87/80
((CH_3_)_3_CCO)_2_O	CeO_2_	--	--	36/0	60/0	75/0
	MnO_2_	--	--	42/0	69/0	84/0

^1^ Pure alumina.

**Table 6:** Activity of 20 wt.% MO_2_/Al_2_O_3_ catalysts (M = Ce or Mn) in ketonization of an equimolar mixture of propanoic and heptanoic anhydrides, T = 698 K; LHSV = 3 cm^3^ g^−1^ h^−1^, V_N2_ = 3 dm^3^ h^−1^.

Active Phase	Yields of Ketones (mol%) ^1,2^			
	C_5_	C_9_	C_13_	Others
CeO_2_	14	68	15	3
MnO_2_	18	59	20	3

^1^ C_5_ = 3-pentanone, C_9_ = 3-nonanone and C_13_ = 7-tridecanone; ^2^ at 698 K conversion of anhydrides, exceeds 98%.

**Table 7:** Cross-ketonization of acetic and diisopropyl isophthalate (13:1 molar ratio) in the presence of 20 wt.% MO_2_/Al_2_O_3_ (M = Ce, Mn or Zr) catalysts. LHSV = 2 cm^3^ g^−1^ h^−1^, V_N2_ = 3 dm^3^ h^−1^.

Active Phase	T	Yield (%)	
	(K)	1,3-DAB ^1^	AP ^2^
-- ^3^	673	2	2
	698	2	3
	723	3	4
MnO_2_	673	6	8
	698	7	9
	723	8	10
ZrO_2_	673	2	3
	698	2	3
	723	3	5
CeO_2_	673	4	6
	698	5	8
	723	8	12
CeO_2_ ^4^	673	2	3
	698	3	4
	723	3	5
CeO_2_ ^5^	673	2	3
	698	3	3
	723	6	9

^1^ 1,3-DAB = 1,3-diacetylbenzene; ^2^ AP = acetophenone; ^3^ pure alumina; ^4^ CeCl_3_·7H_2_O as a precursor; ^5^ (NH_4_)_2_Ce(NO_3_)_6_ as a precursor.

**Table 8:** Cross-ketonization of acetic acid and diisopropyl terephthalate (14:1 mol/mol) in the presence of 20 wt.% MO_2_/Al_2_O_3_ (M = Ce, Mn or Zr) catalysts. LHSV = 2 cm^3^ g^−1^ h^−1^, V_N2_ = 3 dm^3^ h^−1^.

Active Phase	T	Yield (%) ^1^	
	(K)	1,4-DAB ^2^	AP ^3^
MnO_2_	673	4	12
	698	11	51
	723	9	44
CeO_2_	673	10	6
	698	35	17
	723	31	20
CeO_2_ ^4^	673	5	3
	698	9	3
	723	5	4
ZrO_2_	673	3	0
	698	5	0
	723	4	4

^1^ A 97–100% conversion of the appropriate ester has been observed; ^2^ 1,4-DAB = 1,4-diacetylbenzene; ^3^ AP = acetophenone; ^4^ (NH_4_)_2_Ce(NO_3_)_6_ as a precursor.

**Table 9:** Cross-ketonization of acetic acid and various alkyl terephthalates in the presence of 20 wt.% CeO_2_/Al_2_O_3_ catalyst. LHSV = 2 cm^3^ g^−1^ h^−1^, V_N2_ = 3 dm^3^ h^−1^.

Dialkyl Terephthalate	T	Yield (%)	
	(K)	1,4-DAB ^1^	AP ^2^
Dimethyl ^3^	673	6	37
	698	6	36
	723	6	28
Diethyl ^4^	673	14	6
	698	16	8
	723	23	16
Diisopropyl ^4^	673	10	6
	698	35	17
	723	31	20
Diisopropyl ^4,5^	673	2	5
	698	3	6
	723	3	5
Monoisopropyl ^6^	673	--	--
	698	--	--
	723	--	--

^1^ 1,4-DAB = 1,4-diacetylbenzene; ^2^ AP = acetophenone; ^3^ AcOH:ester = 17.6:1 mol/mol; ^4^ AcOH:ester = 14.0:1 mol/mol; ^5^ pure Al_2_O_3_ as catalyst; ^6^ monoisopropyl terephthalate mixed with acetic acid causes the formation of a solid product, which excludes its use in the reaction.

**Table 10:** Time-on-stream tests of cross-ketonization of acetic acid and diethyl or diisopropyl terephthalates (13.2:1 mol/mol) at 698 K in the presence of 20 wt.% CeO_2_/Al_2_O_3_ (ex-Ce(NO_3_)_3_·6 H_2_O) catalyst. LHSV = 2 cm^3^ g^−1^ h^−1^, V_N2_ = 3 dm^3^ h^−1^.

Dialkyl Terephthalate	Time-on-Stream	Yield (%) ^1^	
	(h)	1,4-DAB ^2^	AP ^3^
Diethyl	0.5	22	14
	1	30	13
	2	37	13
	3	39	12
	4	40	14
Diisopropyl	0.5	13	17
	1	35	17
	2	41	18
	3	42	20
	4	40	18

^1^ A 97–100% conversion of the terephthalate esters has been observed; ^2^ 1,4-DAB = 1,4-diacetylbenzene; ^3^ AP = acetophenone.

**Table 11:** Cross-ketonization of acetic and ω-phenyl substituted aliphatic acids (6:1 molar ratio) in the presence of 20 wt.% MO_2_/Al_2_O_3_ (M = Ce or Mn) catalysts. LHSV = 3 cm^3^ g^−1^ h^−1^, V_N2_ = 3 dm^3^ h^−1^.

Active Phase	Acid	T (K)	Yield of Ketone (%)
MnO_2_	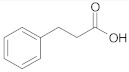	673	48
		698	44
		723	40
	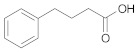	673	49
		698	45
		723	37
CeO_2_	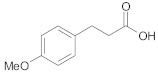	673	47
		698	34
		723	31
	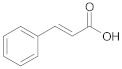	673 ^1^	19
		698	12
		723	2

^1^ AcOH:cinnamic acid = 12:1 (mol/mol).

**Table 12:** Cross-ketonization of acetic acid and various acids containing atoms of N, O or S in heterocycle substituent (6:1 molar ratio) in the presence of 20 wt.% CeO_2_/Al_2_O_3_ catalyst. LHSV = 3 cm^3^ g^−1^ h^−1^, V_N2_ = 3 dm^3^ h^−1^.

Acid	T (K)	Yield of Ketone (%)
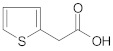	673 ^1^	14
	698	10
	723	5
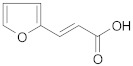	673 ^2^	2
	698	3
	723	2
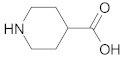	648 ^3^	--
	673	--
	698	--

^1^ Addition of ethyl acetate (0.65:1 mol/mol) to 2-thiopheneacetic acid improves its dissolution in acetic acid; ^2^ molar ratio of AcOH-furylic acid is equal to 14.4:1; ^3^ addition of acetic acid to 4-piperidinecarboxylic acid results in a dramatic increase in viscosity of the resulting mixture, probably due to acid–base reaction, which excludes the use of the mixture in the catalytic test.

**Table 13:** Cross-ketonization of acetic acid and ethyl nicotinate (6:1 molar ratio) in the presence of 20 wt.% MO_2_/Al_2_O_3_ (M = Ce or Mn) catalysts. LHSV = 3 cm^3^ g^−1^ h^−1^, V_N2_ = 3 dm^3^ h^−1^.

Active Phase	T (K)	Ester Conv. (%)	Yield of Ketone (%)
-- ^1^	673	87	3
	698	97	6
	723	99	5
CeO_2_	648	70	3
	673	76	5
	698	86	7
MnO_2_	648	55	2
	673	62	3
	698	77	2

^1^ Pure Al_2_O_3_.

**Table 14:** Cross-ketonization of acetic acid and various ethyl esters of aromatic acids (6:1 molar ratio) in the presence of 20 wt.% CeO_2_/Al_2_O_3_ catalyst. LHSV = 3 cm^3^ g^−1^ h^−1^, V_N2_ = 3 dm^3^ h^−1^.

Ethyl Esters of Acids	T (K)	Ester Conv. (%)	Yield of Ketone (%)
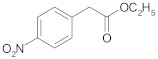	673 ^1^	98–100	0
	698		0
	723		0
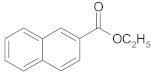	673	59	18
	698	66	27
	723	71	41
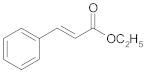	648	75	13
	673	95	8
	698	99	5

^1^ Addition of ethyl acetate (3:1 mol/mol) to ethyl 4-nitrophenylacetate improves its dissolution in acetic acid.

## Data Availability

All data are available in the article.
